# The lipogenic regulator Sterol Regulatory Element Binding Factor-1c is required to maintain peripheral nerve structure and function

**DOI:** 10.1186/2193-1801-4-S1-L45

**Published:** 2015-06-12

**Authors:** Nico Mitro, Gaia Cermenati, Matteo Audano, Silvia Giatti, Maurizio D’Antonio, Emma De Fabiani, Maurizio Crestani, Enrique Saez, Inigo Azcoitia, Guido Cavaletti, Luis-Miguel Garcia-Segura, Roberto C Melcangi, Donatella Caruso

**Affiliations:** DiSFeB, Dipartimento di Scienze Farmacologiche e Biomolecolari, Università degli Studi di Milano, Italy; IRCCS San Raffaele Scientific Institute, DIBIT, Italy; Scripps Research Institute, La Jolla, CA USA; Universidad Complutense de Madrid, Spain; Università degli Studi Milano-Bicocca, Italy; Instituto Cajal, C.S.I.C., Spain

**Keywords:** Peripheral neuropathy, Schwann cells, metabolism

Myelin is a membrane characterized by high lipid content to facilitate impulse propagation. Changes in myelin fatty acid (FA) composition have been associated with peripheral neuropathy [1], but the specific role of peripheral nerve FA synthesis in myelin formation and function is poorly understood. We explored the extent to which lack of the key regulator of FA synthesis as Sterol Regulatory Element Binding Factor-1c (Srebf-1c) could result in the development of peripheral neuropathy. We found that Srebf-1c null mice display a neuropathic phenotype consisting in hypermyelinated small caliber fibers, the result of changes in myelin periodicity. Unexpectedly, transcriptomics and metabolomics revealed activation of peroxisome proliferator activated receptor α (Pparα) signaling in Srebf-1c null peripheral nerve as a result of increased levels of two distinct phosphatidylcholine-based Pparα ligands, PC-C16:0/C18:1 and PC-C18:0/C18:1 [2, 3]. Pparα is a nuclear receptor that directs uptake, utilization and catabolism of FAs [4]. As a consequence of abnormal local Pparα activation, Srebf-1c null peripheral nerve exhibit increased fatty acid utilization, a detrimental condition leading to peripheral neuropathy. Treatment with a Pparα antagonist rescues the neuropathy of Srebf-1c null mice. These findings reveal the importance of FA synthesis to sustain peripheral nerve structure and function.

## References

[1] Cermenati G (2012). J Lipid Res.

[2] Chakravarthy M.V. (2009). Cell.

[3] Liu S (2013). Nature.

[4] Bantubungi K (2012). J Steroid Biochem Mol Biol.

